# Mechanisms of immune evasion of monkeypox virus

**DOI:** 10.3389/fmicb.2023.1106247

**Published:** 2023-02-01

**Authors:** Milad Zandi, Maryam Shafaati, Fatemeh Hosseini

**Affiliations:** ^1^Department of Virology, School of Public Health, Tehran University of Medical Sciences, Tehran, Iran; ^2^Department of Microbiology, Faculty Science, Jahrom Branch, Islamic Azad University, Jahrom, Iran; ^3^Occupational Sleep Research, Baharloo Hospital, Tehran University of Medical Science, Tehran, Iran; ^4^Department of Applied Cell Sciences, School of Advanced Technologies on Medicine, Tehran University of Medical Sciences, Tehran, Iran; ^5^Department of Tissue Engineering and Applied Cell Sciences, School of Advanced Technologies on Medicine, Tehran University of Medical Sciences, Tehran, Iran

**Keywords:** mpox, immune evasion, emerging viral disease, poxviridae, immune system

## Abstract

The mpox (disease caused by the monkeypox virus) epidemic in 2022 provides a good opportunity to study the immune response to mpox. Vaccinia virus-infected monocytes could be recognized by monkeypox virus-specific CD4+ and CD8+ T cells, which produce inflammatory cytokines including IFNγ and TNFα. However, these cells are mostly unable to react to monkeypox virus-infected cells. The monkeypox virus also has no effect on the expression of MHC classes. Cells infected with monkeypox virus can prevent T cells from being activated *via* their T cell receptors. Insensitivity is an MHC-independent strategy for controlling antiviral T cells activation and inflammatory cytokines production. It is likely a critical aspect of virus spread in the infected host. The ability of monkeypox virus to spread efficiently as cell-associated viremia may be explained by the evasion strategies employed by the virus to subvert immunological surveillance by virus-specific T cells.

## Introduction

Many viruses evade the immune system through various mechanisms. Poxviruses can specifically avoid being detected by antiviral chemokines, cytokines, and antigen presentation. Monkeypox virus (MPXV), an orthopoxvirus belonging to the poxviridae family, was first identified as a human pathogen in the Congo region of Africa in 1972. Previous mpox outbreaks (World Health Organization, 2022a) before 2022 were widespread in West and Central Africa, and the mortality rate for MPXV infections was approximately 10%, compared with 30% for variola virus (VARV) infections (Shafaati and Zandi, 2022a). Although mpox causes human disease, it is not rapidly transmitted from one person to another. The discontinuation of the smallpox vaccination, which provides cross-protection against mpox, maybe a key determinant in the 2022 mpox outbreak. The mortality rate in the African clade was 10.6% versus 3.6% for the West African clade (Shafaati and Zandi, 2022b). Due to the current mpox outbreak outside of Africa, the World Health Organization (WHO) updated the names of the clades to I and II, respectively, and warned against stigmas because gay men (MSM) have a higher mortality rate. Even though mpox should not be regarded as a sexually transmitted infection, it’s important to fill information gaps by comprehending how pathogens interacted or competed in previous epidemics (Shafaati et al., 2022).

In 2003, 37 confirmed cases in the Midwestern United States of the first mpox outbreak in the Western Hemisphere. However, this outbreak did not result in any fatalities. The monkeypox virus isolates from the United States were found to be more closely related to the less virulent West African monkeypox virus strains than to the more virulent Central African strains by DNA sequencing (Huhn et al., 2005). This may explain why the 2003 mpox outbreak did not result in human deaths. Given the eradication of smallpox and the end of the smallpox vaccination campaign in humans, there is growing concern that MPXV and VARV could be used as biological weapons (Fine et al., 1988; Shafaati et al., 2022). As of December 13, 2022, mpox outbreaks in 110 countries had led to 65 mortality and 82,628 confirmed cases, according to WHO records (World Health Organization, 2022b).

We claimed in our earlier paper (Shafaati et al., 2022) that the dynamics of 2022 mpox transmission are still unknown. Given that time is of the essence in an epidemic, the WHO’s delay in releasing a statement on the recent spread of mpox outside of Africa can be regarded as disregarding an important portion of specific groups of people with certain sexual orientations, as well as regions of the world like Africa. One of the “microbiome exchange” pathways is sexual contact. Although mpox is not a sexually transmitted infection, contact with gay men (MSM) has been confirmed as the most recent transmission route (Bragazzi et al., 2022). In addition to the cessation of smallpox vaccination, which resulted in the creation of a susceptible population throughout the world, other factors, such as the loss of immunization, the increase in risky behaviors and changes in human lifestyle, the rise in the number of people with immune deficiencies, changes in the properties of the virus, and an increase in animal hosts, have all contributed to the rise in the number of people with immune deficiencies. All have improved the mpox ability to spread from person to person since HIV-gay-related immunodeficiency (GRID) is significant in immune-deficiency conditions (Einav and Cleary, 2022; Otero and Sanjuan, 2022).

All verified MPXV and HIV cases had bacterial infections, vaginal ulcers, or skin rashes. As a result, this may be the cause of the evolutionary dynamics that aid in the development and reemergence of viruses caused by human activity and behavior (Ogoina et al., 2020).

Considering that the current human population has little or no immunity to smallpox, this is very problematic. Although the vaccinia virus (VACV; smallpox vaccine) is 85% effective in preventing disease, it is highly unlikely that it will be used as a pre-exposure vaccine because of the adverse side effects of vaccination (Arndt et al., 2015; Shafaati and Zandi, 2022b). For these reasons, there is an increasing need for evidence on how monkeypox virus might infiltrate the immune system and infect humans.

### Evasion of the innate immune in orthopoxviruses

Most of the information on orthopoxviruses innate immune evasion comes from research on VACV and the suppression of the host antiviral immune response. The antiviral immune response, mediated by cellular IFN, is strongly inhibited by the E3L gene of VACV (Weaver and Isaacs, 2008). To prevent activation of known pattern recognition receptors such as protein kinase R, MDA-5, RIG-I, and OAS, the E3 protein can bind double-stranded RNA and secrete it from them. An N-terminal Z-nucleic acid binding domain and a C-terminal dsRNA-binding domain form the two conserved domains of the VACV-E3 protein (Xu et al., 2008). In a mouse model and *in vitro*, wild-type virus pathogenesis depends on these two domains. The PKR pathway can be completely inhibited only when the N-and C-terminal domains of the VACV-E3 protein are present (Arndt et al., 2015; Shafaati and Zandi, 2022a).

Two VACV recombinants, VACV-E3L83N (which has the first 83 amino acids of the N terminus of E3L removed) and VACV-E3L37N (which encodes only the smaller of the two proteins [p20] translated from E3L mRNA), have been shown in *in vitro* studies to have similar host range and IFNr phenotype to wild-type VACV in most cells in culture (Edghill-Smith et al., 2005). However, VACV-E3L83N and VACV-E3L37N were 100–1,000 times less pathogenic than wild-type VACV in an animal model. Phosphorylation of the alpha subunit of eukaryotic translation initiation factor 2 (eIF2) was observed in cell lines infected with either virus at a late times point (9 h postinfection), but not in wild-type VACV-infected HeLa cells (Shafaati and Zandi, 2022c).

### Monkeypox virus and mechanisms of immune evasion

**Figure 1:**
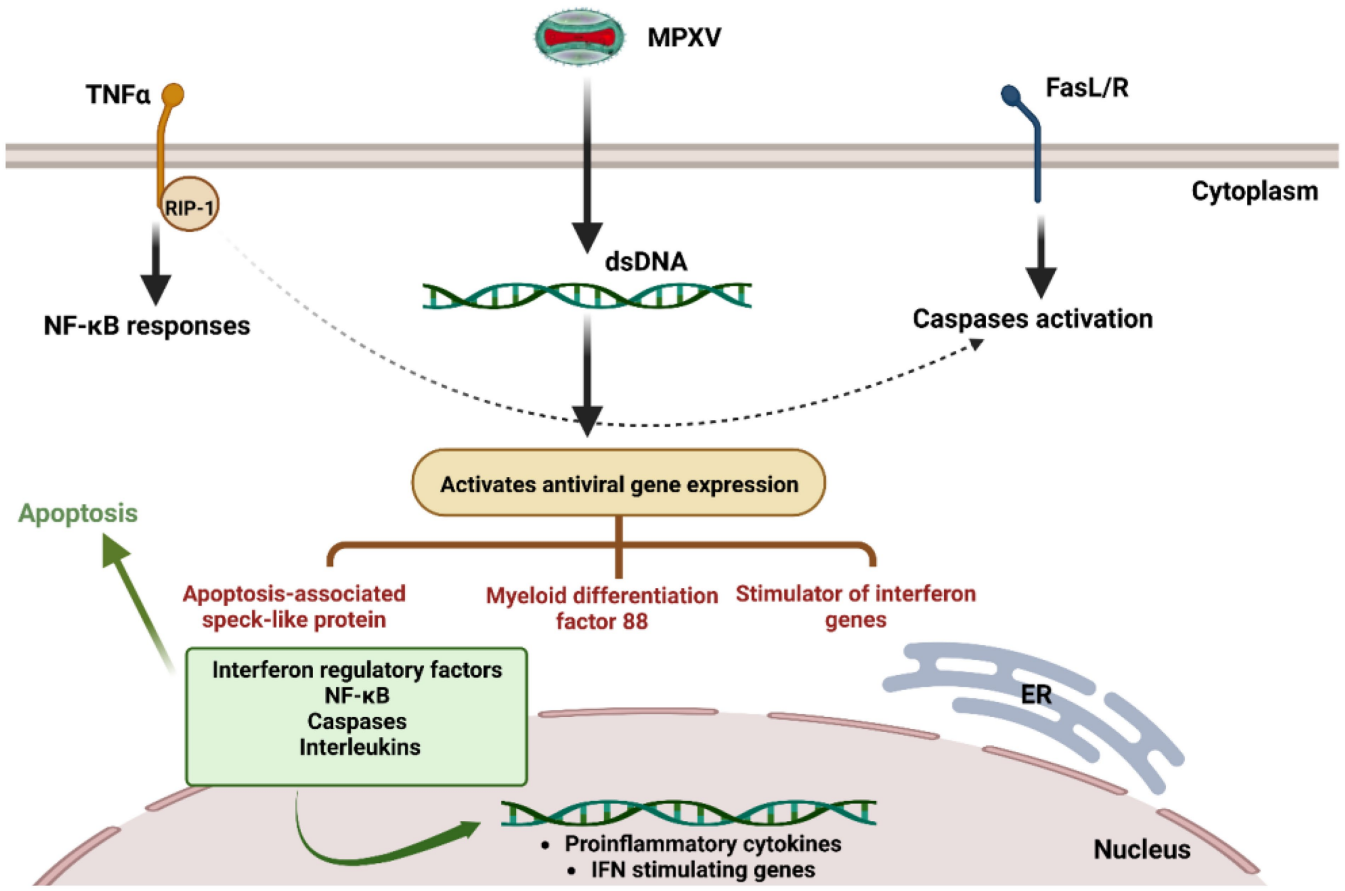
Suggested monkeypox virus immune evasions. During infection, the MPXV can escape from a series of downstream and upstream elements that cause the production of interferons, cytokines, and interleukins for the antiviral immune response.

Genomic studies revealed sequence similarities between MPXV and VACV. With 92% nucleotide and 88% protein sequence similarity, the F3 protein of MPXV and E3 protein of VACV are homologous to each other (Arndt et al., 2015). According to the MPXV genome sequence, the C-terminal dsRNA-binding domain of the F3 protein is intact and functional, but the first 37 amino acids of the Z-NA binding domain are missing (Yuwen et al., 1993; Langland and Jacobs, 2004). According to Arndt et al., the MXPV genome encodes a modified E3 homolog. MPXV is identical to wild-type VACV in terms of IFNr and host range and can suppress the cellular antiviral immune response more effectively than a VACV mutant with an identical N-terminal deletion in E3. These results suggest that MPXV can prevent symptoms caused by the absence of an N-terminal region in its E3 homolog (Resch et al., 2007). The failure of VACV to completely inhibit PKR or restore replication in cells in culture when F3l is replaced by E3L indicates that inhibition of the absence of an N-terminal domain of F3 is extragenic (Arndt et al., 2015; Lum et al., 2022; Figure 1).

In addition, cowpox virus (CPV), another orthopoxvirus, has been shown to inhibit intracellular transport of MHC class I, a process associated with CPV evasion of the CD8+ T-cell antiviral responses (Komiyama et al., 1994). CPV ORF203 has been shown to maintain MHC class I at ER. However, MPXV encoding a near-homologous CPV ORF203 cannot downregulate MHC Class I. Instead, it can employ an evasion strategy to block activation of CD4+ and CD8+ T-cells after cognate contacts with MPXV-infected cells (Shchelkunov, 2012). This strategy of suppressing local T-cell responses could avoid systemic immunosuppression while hiding the viral reservoir from immunological surveillance. By using a specific mechanism to bypass the immune system, MPXV can avoid both CD4+ and CD8 + T-cell responses, which are antiviral (Song et al., 2013). The inability of T-cells to respond is an MPXV-infected cell-dependent and MHC-independent phenomenon. This implies that MPXV encodes an immunomodulator that blocks antiviral T-cell responses that are activated either directly or indirectly by the host. This inhibition is likely critical for viral pathogenicity as well as for systemic transmission of cell-associated viruses (Lum et al., 2022). It may be possible to understand why mpox can spread rapidly as a type of cell-associated viremia by studying the mechanism by which virus-specific T-cells bypass immune surveillance. Neutralizing antibodies to orthopoxviruses are critical for protection against severe infections, and systemic proliferation in circulating monocytes may also protect the virus from humoral immune responses, explaining why vaccinated monkeys are protected. Memory T-cells specific for orthopoxviruses do not provide protection against lethal mpox in the absence of neutralizing antibodies (Shafaati and Zandi, 2022c). After recovery from mpox, individuals may activate antiviral T-cell responses. Although there is an immune evasion mechanism that strongly inhibits T-cells from recognizing monkeypox virus. The endogenous T-cell response is triggered indirectly by infected monocytes through the presentation of an alternative antigen and/or cross priming (Cho and Wenner, 1973; Slifka, 2004; Edghill-Smith et al., 2005). Neutralizing antibodies must also be present to prevent virulent orthopoxvirus infections. Circulating monocytes, through which virus spreads throughout the body, may protect it from the effects of virus-specific humoral immunity (Hammarlund et al., 2008).

Immunity to mpox is typically determined by studying interactions between various orthopoxviruses like the vaccinia virus (VACV). It has been proposed that the poxvirus antigen on neutrophils and monocytes significantly determines mpox mortality. It was also found that primary human M2-like macrophages support VACV replication and transmission. After becoming infected, these primary macrophages produced actin tails, cell junctions, lamellipodia, and branching structures related to VACV virions, indicating that these cells may aid in the spread of the MPXV (Hammarlund et al., 2008; Rubins et al., 2011). A similar strategy could be used by a monkeypox virus to evade the host’s immunity. Because several innate immune cells, including monocytes or macrophages, neutrophils, natural killer cells, dendritic cells, and innate lymphoid cells, have not yet been identified. A smallpox vaccination also provides cross-protection against mpox (Zandi et al., 2022). There is a significant degree of sequence similarity among orthopoxviruses, especially in immunologically significant proteins, leading to numerous shared immune epitopes. T cell responses (CD4+ and CD8+ T cells) can identify epitopes on a wide range of viral proteins. People with mpox may get post-exposure prophylaxis, such as vaccination, immune globulin, or antiviral therapies. Jynneos and IMVANEX vaccines are used to provide protective immunity against mpox (Shafaati and Zandi, 2022c).

## Conclusion

Since the end of smallpox vaccination, the prevalence of mpox has increased. As a member of the poxvirus family, monkeypox virus has the ability to manipulate both the innate and adaptive immune systems. It is important for the development of vaccines and antiviral therapeutics to know the strategies of monkeypox virus to evade immune.

## Author contributions

MZ conceived the idea and developed concepts, and wrote and edited the paper. MS and FH wrote, edited and revised the paper. All authors contributed to the article and approved the submitted version.

## Conflict of interest

The authors declare that the research was conducted in the absence of any commercial or financial relationships that could be construed as a potential conflict of interest.

## Publisher’s note

All claims expressed in this article are solely those of the authors and do not necessarily represent those of their affiliated organizations, or those of the publisher, the editors and the reviewers. Any product that may be evaluated in this article, or claim that may be made by its manufacturer, is not guaranteed or endorsed by the publisher.
